# Decreased Fast Ripples in the Hippocampus of Rats with Spontaneous Recurrent Seizures Treated with Carbenoxolone and Quinine

**DOI:** 10.1155/2014/282490

**Published:** 2014-09-03

**Authors:** Consuelo Ventura-Mejía, Laura Medina-Ceja

**Affiliations:** Laboratory of Neurophysiology and Neurochemistry, Department of Cellular and Molecular Biology, Centro Universitario de Ciencias Biológicas y Agropecuarias (CUCBA), University of Guadalajara, Camino Ing. R. Padilla Sánchez 2100, Las Agujas, Nextipac, Zapopan, JAL, CP 45110, Mexico

## Abstract

*Background*. In models of temporal lobe epilepsy and in patients with this pathology, high frequency oscillations called fast ripples (FRs, 250–600 Hz) can be observed. FRs are considered potential biomarkers for epilepsy and, in the light of many *in vitro* and *in silico* studies, we thought that electrical synapses mediated by gap junctions might possibly modulate FRs *in vivo*. *Methods*. Animals with spontaneous recurrent seizures induced by pilocarpine administration were implanted with movable microelectrodes in the right anterior and posterior hippocampus to evaluate the effects of gap junction blockers administered in the entorhinal cortex. The effects of carbenoxolone (50 nmoles) and quinine (35 pmoles) on the mean number of spontaneous FR events (occurrence of FRs), as well as on the mean number of oscillation cycles per FR event and their frequency, were assessed using a specific algorithm to analyze FRs in intracranial EEG recordings. *Results*. We found that these gap junction blockers decreased the mean number of FRs and the mean number of oscillation cycles per FR event in the hippocampus, both during and at different times after carbenoxolone and quinine administration. *Conclusion*. These data suggest that FRs may be modulated by gap junctions, although additional experiments *in vivo* will be necessary to determine the precise role of gap junctions in this pathological activity associated with epileptogenesis.

## 1. Introduction

Epilepsy is a neuronal disorder that is characterized by the abnormal, continued discharge and hypersynchronous activity of neurons [1]. The existing models of epilepsy provide good tools to study the basic mechanisms by which seizures are generated, and the model of temporal lobe epilepsy (TLE) induced by pilocarpine or kainic acid (KA) simulates most of the characteristics of this pathology [2–4]. In this TLE model, high frequency oscillations known as fast ripples (FRs, 250–600 Hz) have been observed, as in tetrodotoxin and tetanus toxin models of epilepsy [5–7], in computational models* in silico* [8, 9] and in patients with TLE [2, 10, 11].

FRs can be evoked by electrical stimulation of the same brain areas where they occur spontaneously and they reflect bursts of population spikes from synchronously firing principal cells in relatively small areas of the hippocampus (HIP, 1 mm^3^) [12–14]. According to electrophysiological studies in animals with spontaneous recurrent seizures, these are areas that contain groups of pathologically interconnected neurons [2, 15, 16]. However, FRs have only been recorded in the HIP and entorhinal cortex (EC) ipsilateral to the lesion during slow wave sleep and immobility, albeit for periods ranging from days to months [2, 17]. In addition, various studies have confirmed that the onset of focal seizures coincides with fast ripple (FR) activity [10, 18, 19]. Accordingly, FRs are considered to be potential biomarkers for epilepsy as they can be recorded both before seizure onset and during seizure activity [3, 16].

Electrical synapses enable electrical activity to be synchronized between neurons and they permit the spread of depolarization (excitation) or hyperpolarization (inhibition) across a particular neural network [20]. Several studies have highlighted the involvement of electric coupling in epileptiform activity, both in* in vitro* and* in vivo* models [19, 20]. Moreover, the loss of electrical coupling provoked by gap junction blockers (e.g., carbenoxolone [CBX], a nonspecific blocker of connexins, or quinine, a dose-dependent blocker of connexin 36) [21, 22] or through a deficiency in connexins, the structural proteins that form gap junctions, produces antiepileptic effects [23–30]. Indeed, recent* in silico* studies [31] supported the participation of gap junctions localized in axons in the generation and persistence of gamma and FR activity.

Based on this information, we have considered the possible involvement of electrical synapses mediated by gap junctions in the modulation of FRs in animals experiencing spontaneous recurrent seizures. For this propose, we evaluated the effects of CBX and quinine on the mean number of spontaneous FR events (FR occurrence), as well as the mean number of oscillation cycles per FR event and frequency.

## 2. Methods

### 2.1. Model of Temporal Lobe Epilepsy Induced by Intracerebroventricular Pilocarpine Administration

Male Wistar rats (190–200 g) were housed individually in cages in a temperature-controlled room (22 ± 2°C), on a 12 h light/dark cycle (lights on from 7:00 a.m. to 7:00 p.m.) and with* ad libitum* access to food and water. All experimental procedures were designed to minimize animal suffering and the total number of animals used. The protocols used were in accordance with the Rules for Research in Health Matters (Mexican Official Norms NOM-062-ZOO-1999, NOM-033-ZOO-1995) and they were approved by the local Animal Care Committee.

To induce acute status epilepticus (SE) [32], rats were anesthetized with isofluorane (Sofloran, PISA, Laboratories, Mexico) in 100% oxygen and secured in a Stoelting stereotaxic frame with the incisor bar positioned at −3.3 mm. A hole was drilled in the skull above the right lateral ventricle at the following stereotaxic coordinates relative to bregma: AP −4.1 mm, L −5.2 mm, and V 7 mm. A single dose of pilocarpine hydrochloride (1.2 mg/*μ*L, total volume 2 *μ*L; Sigma-Aldrich, USA) was injected through a needle connected to an injection pump (flow rate: 1 *μ*L/min; Stoelting Co., IL, USA). After recovery, the animals returned to their cages for observation, convulsive behavior was scored according to the Racine scale [33], and animals with a score of 4/5 were considered to exhibit SE. After 90–120 minutes, SE was abolished by administering diazepam (5–10 mg/kg, i.p.) to increase their survival and, when necessary, two doses of diazepam were administered. The convulsive behavior of all the animals after pilocarpine injection was monitored visually and 15 days after SE induction the rats were video monitored for 24 hours every day, scoring their spontaneous recurrent seizures. Animals exhibiting spontaneous recurrent seizures were selected for microelectrode implantation.

### 2.2. Surgery

Control rats were anaesthetized as indicated above and they were positioned in a stereotaxic frame such that lambda and the bregma were in the same horizontal plane. In the control group, fixed recording microelectrodes, consisting of pairs of tungsten wires (60 *μ*m in diameter) with a 1.5 mm vertical tip separation, were implanted in the right anterior (RAH: AP −3.5 mm; ML 2.00 mm; DV 4.0 mm) and posterior hippocampus (RPH: AP −5.0 mm relative to bregma; ML 5.0 mm; DV 5.5 mm). In addition, two stainless steel screws were driven into the bone, one above the bregma and one above the cerebellum, which served as indifferent and ground electrodes, respectively. Finally, a stainless steel guide cannula (0.5 mm internal diameter) was implanted through a hole drilled in the skull and positioned in the region of the right EC (AP −8.00 mm, ML 4.0 mm, DV 5.0 mm) in order to insert a needle for the injection of various chemical agents (CBX, quinine, and saline solution). This arrangement of microelectrodes was placed on a pin connector and, along with the guide cannula, fastened to the skull with dental cement. For experimental groups, an arrangement of eight microelectrodes with the same characteristics as those described above was mounted in a mobile device and implanted into the right region of the hippocampus (RAH, AP −3.5 mm, ML 3.0 mm, DV 2.5 mm; RPH, AP −5.0 mm, ML 3.0 mm, DV 2.5 mm) in order to move this device to different depths of the hippocampus and easily detect FRs in experiments carried out in freely moving rats.

### 2.3. Drug Administration

To determine the involvement of electrical synapses, we used three control (*n* = 3 rats each one) and three experimental groups of animals (*n* = 6 each one) with recurrent spontaneous seizures, the latter receiving CBX (50 mM, final dose 50 nmoles), quinine (35 mM, final dose 35 pmoles), or the vehicle alone (NaCl 0.9%; Sigma Chemical Co. St. Louis, MO, USA). These drugs were administered through an injection needle placed into the guide cannula (0.2 *μ*L/min flow for 5 minutes) and using a microsyringe mounted to a microinjection pump (WPI, Fl. USA).

### 2.4. Intracranial EEG Recordings and Analysis

Intracranial EEG activity was recorded in freely moving rats. Five 4-channel MOSFET small amplifiers were attached to the cable connector to eliminate movement artifacts. Hippocampal electrical activity was recorded on a polygraph with eight amplifiers (Model 7D, Grass Technologies, RI, USA) at a bandwidth of 0.1 to 3 kHz and with a sensitivity of 75 *μ*V/cm per channel. The sampling rate was set at 5 kHz/channel with 12-bit precision using an iMac A1048 (Apple, USA) and MP150 software system (BIOPAC Systems, CA, USA). The basal electrical activity of each control group was analyzed, and the amplitude and frequency averaged over a 15 min recording period. In contrast, once recorded, EEG traces from all recordings of experimental animals were converted to MATLAB readable files to process and identify the FRs using an algorithm designed specifically for this purpose [34–36]. Accordingly, each of the signals selected was passed through a 100–650 Hz band-pass filter using the Hamming method with 60 coefficients. To identify FRs, the filtered signal was processed using the root mean square (RMS) in a sliding window of 3 ms and through its successive values. Values that were over 5 times the standard deviation of the mean value of the filtered signal and that lasted at least 6 ms were considered possible FR events. A second criterion was also used for FR classification, whereby the changes in the approximate entropy of the signal were analyzed [36, 37] and those that were over twice the mean value were considered putative FRs. Signals that fulfilled these two criteria were processed with the fast Fourier transform (FFT) to calculate their frequency in Hz. In addition, the results from the automated FRs detection algorithm were manually inspected. The FRs parameters evaluated were the mean number of spontaneous FR events (occurrence of FRs per 15 min) and the mean number of oscillation cycles per FR event, as well as the frequency before, during, and after CBX or quinine administration.

### 2.5. Histological Evaluation

After each experiment the animals were anesthetized with sodium pentobarbital and perfused transcardially with 100 mL of normal saline (0.9%) in 0.12 M buffer/CaCl_2_, followed by 300 mL of 4% paraformaldehyde in 0.12 M buffer/CaCl_2_ (pH 7.3). The animal's brain was then removed, and coronal sections (50 *μ*m thick) were obtained and stained with cresyl violet to confirm the correct positioning of the guide cannula and microelectrodes. Only animals that showed a correct implantation of cannula and microelectrodes were included in the present work.

### 2.6. Statistical Analysis

In the control groups the data are expressed as the mean ± SEM of the amplitude and frequency of electrical activity recorded in the RAH, LAH, RPH, and LPH (right/left anterior/posterior hippocampus). Significant differences between control groups were obtained by an analysis of variance (ANOVA, one-way) followed by Tukey's test. The data from the experimental groups are expressed as the mean ± SEM of each parameter evaluated before, during, and after CBX or quinine administration. Significant differences were analyzed by ANOVA followed by Dunnett's post hoc test, with *P* values <0.05 considered significant.

## 3. Results

### 3.1. Behavior and Analysis of Intracranial EEG Recordings in Control Animals

**Figure 1:**
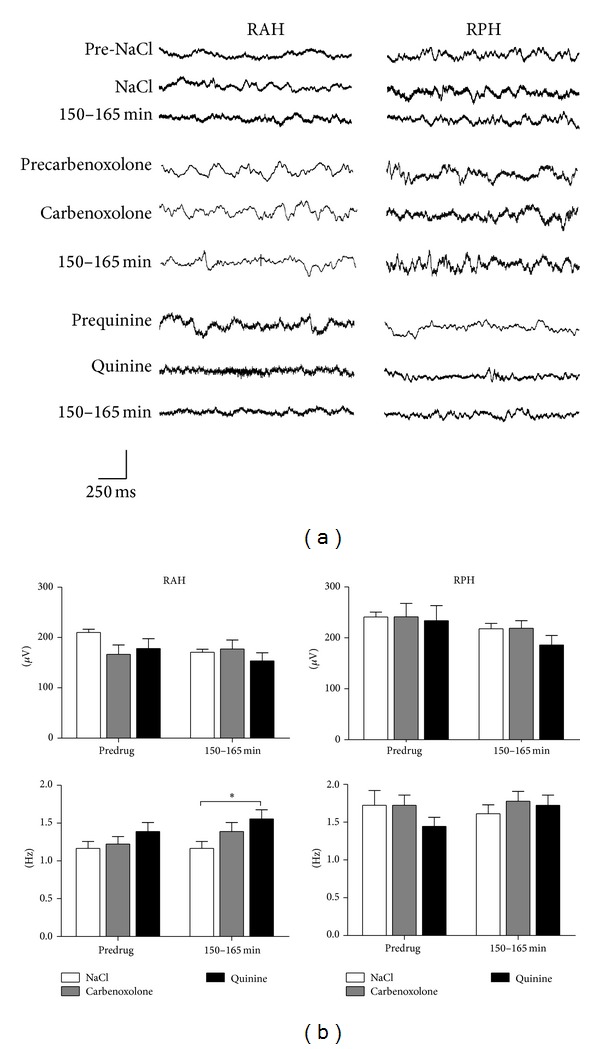
Representative intracranial EEG recordings from the different brain regions studied in three different rats from the NaCl, CBX, and quinine control groups (*n* = 3 each one). The lower graphs show the mean amplitude (*μ*V ± SEM) and frequency (Hz ± SEM) of the electrical activity observed before (PRE-DRUG) and 150–165 min after drug administration. The *y*-axis calibration bar corresponds to the amplitude: 0.4 mV for NaCl, 1 mV for CBX, and 0.5 mV for quinine groups (*P < 0.05).

Animals from the three control groups showed normal behavior before, during, and after drug administration into the EC (CBX 50 nmoles, quinine 35 pmoles, and NaCl 0.9%). In these animals normal grooming, chewing movements, exploratory behavior, and sleep periods were observed. The analysis of the intracranial EEG recordings from these control rats revealed a low amplitude and frequency of electrical activity (Figure 1) in all the regions analyzed, both before and after drug administration. The only significant difference observed was in the frequency parameter in the 150–165 min period analyzed between animals from the control groups treated with NaCl and quinine (1.16 ± 0.09 Hz versus 1.55 ± 0.12 Hz, resp., Figure 1).

### 3.2. Effect of Carbenoxolone on the Fast Ripples Observed in Animals with Spontaneous Seizures

In terms of their behavior, the rats that received CBX showed vibrissae movements, and while they remained in a state of sleep for most of the analysis, spontaneous seizures were observed in some animals at level 5 of the Racine scale during the experiments.

**Figure 2:**
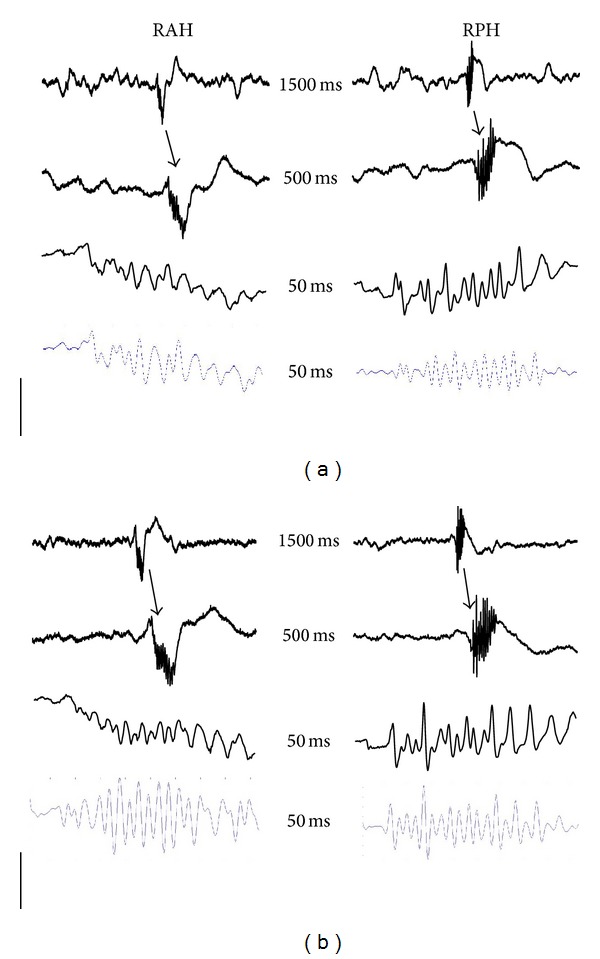
Representative intracranial EEG recordings taken before drug administration from the different brain regions studied in two rats from the experimental groups (*n* = 6 each one) with CBX (a) and quinine (b), in which spontaneous fast ripples (FRs) were observed simultaneously: right anterior hippocampus (RAH) and right posterior hippocampus (RPH). An EEG trace of 1.5 s with a spontaneous FR (arrow in the first trace) followed by extended EEG traces of the same activity. The last EEG trace is with an 80 Hz filter. The numbers in the centre correspond to the time of each EEG trace and the *y*-axis calibration bar corresponds to the amplitude: 1 mV for the first and 0.4 mV for the rest of the EEG traces.

**Figure 3:**
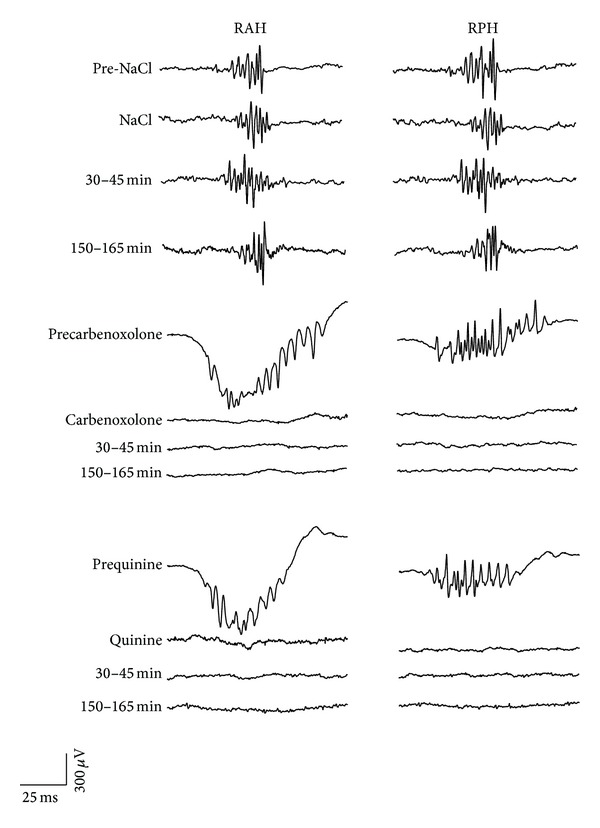
Representative intracranial EEG recordings from the different brain regions studied and obtained from three different rats in the NaCl, CBX, and quinine experimental groups (*n* = 6 each one) in which spontaneous fast ripples (FRs) were observed: right anterior hippocampus (RAH) and right posterior hippocampus (RPH) before, during, and at different times after drug administration. Note the effects of CBX and quinine on the FRs.

**Figure 4:**
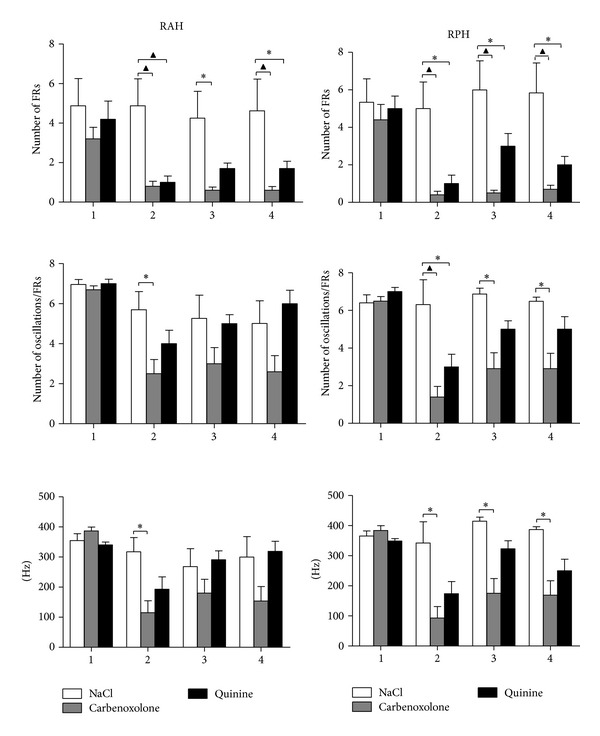
Graphs show the mean number of spontaneous FR events (spontaneous FRs ± SEM) and the oscillation cycles per spontaneous FR, as well as the mean frequency (Hz), before (1), during (2), and at different times after NaCl (NaCl, 0.9%), CBX (50 nmoles), and quinine (35 pmoles) administration (3, 30–45 min; 4, 150–165 min) in the regions studied: right anterior hippocampus (RAH) and right posterior hippocampus (RPH). A significant decrease in FR number was provoked by CBX and quinine administration in the RAH and RPH (*P < 0.05; ^▲^ *P* < 0.001).

When the intracranial EEG recordings from experimental animals (*n* = 6) with spontaneous and recurrent seizures were analyzed, spontaneous FRs were evident in all the regions registered (Figure 2(a)). The administration of CBX (50 nmoles) produced a significant decrease in the mean number of FRs in the RAH and RPH compared to those that received NaCl (Figures 3 and 4), both during and at different times after CBX administration (RAH: during 4.8 ± 1.4 versus 0.8 ± 0.2; after 30–45 min, 4.2 ± 1.4 versus 0.6 ± 0.1; after 150–165 min 4.6 ± 1.7 versus 0.6 ± 0.2. RPH: during, 5.0 ± 1.5 versus 0.4 ± 0.1; after 30–45 min, 6.0 ± 1.6 versus 0.5 ± 0.1; after 150–165 min, 5.8 ± 1.7 versus 0.7 ± 0.2). By contrast, the mean number of oscillation cycles per FR event decreased in the RPH during and at different times after CBX administration (during 6.3 ± 1.4 versus 1.4 ± 0.5; after 30–45 min 6.8 ± 0.3 versus 2.9 ± 0.8; after 150–165 min 6.48 ± 0.2 versus 2.9 ± 0.8), yet only during CBX administration in the RAH (5.69 ± 0.9 versus 2.5 ± 0.7). The mean frequency of FRs also decreased in the RPH compared with the rats that received NaCl alone, both during and at different times after CBX administration (during 342.1 ± 77.3 versus 93 ± 39; after 30–45 min 414.6 ± 14.5 versus 175 ± 51; after 150–165 min 386.6 ± 10.5 versus 169 ± 49), yet only during CBX administration in the RAH (317.2 ± 51 versus 115 ± 41 Hz).

### 3.3. Effect of Quinine on the Fast Ripples Observed in Animals with Spontaneous Seizures

The animals that received quinine exhibited vibrissae movements and they remained in a state of sleep for most of the analysis, although level 5 spontaneous seizures of the Racine scale were observed in some of these animals.

Like the animals that received CBX, spontaneous FRs were evident in all the regions analyzed when rats received quinine (Figure 2(b)), although there were fewer FRs during quinine administration in the RAH and 150–165 min after quinine administration (Figures 3 and 4: during 4.8 ± 1.4 versus 1.0 ± 0.3; after 150–165 min 4.6 ± 1.7 versus 1.7 ± 0.4). By contrast, there were fewer FRs in the RPH at all the EEG recording periods analyzed (during 5.0 ± 1.5 versus 1.0 ± 0.5; after 30–45 min 6.0 ± 1.6 versus 3.0 ± 0.7; after 150–165 min 5.8 ± 1.7 versus 2.0 ± 0.5). While in the RPH a significant decrease in the mean number of oscillation cycles per FR was observed only during quinine administration (6.3 ± 1.4 versus 3.0 ± 0.7) there was no difference in the mean frequency of FRs in the R AH and R PH.

## 4. Discussion

In the present study, no differences were found in the behavior observed in animals from control groups and electrical activity was characterized by the presence of slow physiological waves of low amplitude and frequency, although a slight increase in the frequency was observed between the saline solution control animals and those that received quinine. However, this difference could be due to different behavioral states of control animals considering that these animals were observed quiet and asleep, 150–165 min after drug administration. Moreover this difference did not appear to be physiologically relevant for our study given that the frequency of both groups did not exceed 2 Hz.

In this study spontaneous FRs were detected in all the hippocampal brain regions analyzed from animals with spontaneous and recurrent seizures induced by the i.c.v. administration of pilocarpine, animals chosen after analyzing their intracranial EEG recordings with an algorithm exclusively designed to detect FRs. The movable recording microelectrodes that we used in the experimental animals were situated at different depths of hippocampal regions in order to detect FRs, which are generated in relatively small areas (1 mm^3^) [15, 16]. The mean number and frequency of spontaneous FRs observed here were similar to those described previously in animals administered unilateral hippocampal injections of KA [2, 15]. Moreover, unlike other studies, spontaneous FRs were observed during immobile, waking, and sleeping states of the animals, although periods of slow-wave sleep were not specifically analyzed, when FRs are more frequent [15, 38]. There have been many studies into FRs and their probable mechanism of generation [5, 12–16, 39], considering that FRs may be potential biomarkers for epilepsy and useful to detect candidate areas for resection [40]. Nevertheless, there are no* in vivo* studies about factors that could modulate FRs by electrical synapses.

We found that the nonspecific blocker of gap junctions, CBX, decreased the mean number of FRs in the RAH and RPH, as well as the mean number of oscillation cycles per FR event, and the frequency in the RPH during and at different times after CBX administration. These data indicate a probable role of gap junctions in modulating FRs, although there is no data to date from studies* in vivo* with which the results of present study can be compared. Nevertheless, similar results were reported in some* in vitro* studies. In a study carried out on slices from epileptic patients, CBX (0.2 mM) provoked a 50% decrease in FRs [31] as in other study [9], while ripple activity (150–250 Hz) was also blocked by the gap junction blocker halothane [41, 42]. Likewise, quinine decreased the mean number of FRs in the RAH and RPH during and at different times after its administration, while the mean number of oscillation cycles per FR event only decreased in the RPH, suggesting only a partial modulatory effect of quinine on FRs. This effect could be due to quinine blocking gap junctions formed only by Cx36, which are found in neurons, and we speculate that gap junctions formed by different connexins are required for FR activity, as observed here through the effects of the nonspecific blocker, CBX.

There are currently no* in vivo* studies to compare our results regarding the effects of quinine, although there is significant data supporting the important role of gap junctions in epileptiform activity in different* in vitro* and* in vivo* models. Indeed, the disruption of electrical coupling induced by gap junction blockers or through a deficiency in certain Cx proteins that make up gap junctions has antiepileptic effects [20, 24, 27–30]. Similarly, a role for Cx36 was shown in the generation of high frequency oscillations (100–200 Hz) and epileptiform field bursts recorded in slices from the hippocampus of deficient mice [26]. other* in silico* study has demonstrated the importance of axoaxonal gap junctions in FR generation [31]. These data support our data regarding the modulation of FRs by gap junctions, although we cannot rule out the influence of other factors that could modulate FRs, such as the chemical neurotransmission. In relation to this, serotonin was found to reduce electric coupling through protein kinase C, via IP_3_/Ca^2+^ [43], and probable through 5-HT2 receptor activation [44]. In addition, the frequency of FRs was higher during slow wave sleep, a period in which serotonin levels are low in the model of chronic seizures induced by KA [2, 11, 15]. Indeed, a modulatory effect of serotonin was observed on electric synapses in weakly electrical coupled neural networks of the* Helisoma *ganglia [45]. In conjunction with our data regarding serotonin and FR modulation [46], these facts also suggest a possible influence of serotonin neurotransmission on gap junction activity and, hence, in the modulation of FRs.

The CBX and quinine have been clinically used against malaria and ulcers but they have not been completely tested for their anticonvulsant effects in patients; therefore it is necessary to carry out more* in vivo* experiments to find the role of gap junctions in the FRs modulation in order to consider this strategy as a possible clinical implication.

## 5. Conclusions

Through the present data, we conclude that gap junctions could exert a modulatory effect on FRs in the hippocampus of rats with spontaneous seizures induced by pilocarpine. While these effects probably occur through gap junctions formed by different connexins, we cannot rule out the possible participation of chemical synapses in FR modulation, such as that of serotonin neurotransmission. Finally, it is necessary to perform additional* in vivo* experiments to determine the precise role of gap junctions in the modulation of FRs associated with epilepsy in the hippocampus.
